# Signal mingle: Micropatterns of BMP-2 and fibronectin on soft biopolymeric films regulate myoblast shape and SMAD signaling

**DOI:** 10.1038/srep41479

**Published:** 2017-01-30

**Authors:** Vincent Fitzpatrick, Laure Fourel, Olivier Destaing, Flora Gilde, Corinne Albigès-Rizo, Catherine Picart, Thomas Boudou

**Affiliations:** 1 CNRS, LMGP, F-38000 Grenoble, France; 2 University Grenoble Alpes, LMGP, F-38000 Grenoble, France; 3 INSERM U1209, Institute for Advanced Biosciences, Institut Albert Bonniot, 38700 La Tronche, France; 4 CNRS UMR5309, Institute for Advanced Biosciences, Institut Albert Bonniot, 38700 La Tronche, France; 5 Université Grenoble Alpes, Grenoble, France.

## Abstract

*In vivo*, bone morphogenetic protein 2 (BMP-2) exists both in solution and bound to the extracellular matrix (ECM). While these two modes of presentation are known to influence cell behavior distinctly, their role in the niche microenvironment and their functional relevance in the genesis of a biological response has sparsely been investigated at a cellular level. Here we used the natural affinity of BMP-2 for fibronectin (FN) to engineer cell-sized micropatterns of BMP-2. This technique allowed the simultaneous control of the spatial presentation of fibronectin-bound BMP-2 and cell spreading. These micropatterns induced a specific actin and adhesion organization around the nucleus, and triggered the phosphorylation and nuclear translocation of SMAD1/5/8 in C2C12 myoblasts and mesenchymal stem cells, an early indicator of their osteoblastic trans-differentiation. We found that cell spreading itself potentiated a BMP-2-dependent phosphorylation of SMAD1/5/8. Finally, we demonstrated that FN/BMP-2-mediated early SMAD signaling depended on LIM kinase 2 and ROCK, rather than myosin II activation. Altogether, our results show that FN/BMP-2 micropatterns are a useful tool to study the mechanisms underlying BMP-2-mediated mechanotransduction. More broadly, our approach could be adapted to other combinations of ECM proteins and growth factors, opening an exciting avenue to recreate tissue-specific niches *in vitro.*

Due to their physiological relevance, bone morphogenetic proteins (BMPs) are widely studied for orthopedic clinical applications to enhance the healing of large bone defects^1^,^2^, as well as for developing new strategies in bone tissue engineering^3^,^4^,^5^. BMPs are indeed highly potent growth factors (GFs) that play a crucial role in morphogenesis and tissue homeostasis during embryonic development and until adulthood^6^,^7^. In particular, BMP-2 promotes the differentiation of mesenchymal stem cells (MSCs) and osteoblasts toward osteocytes^8^,^9^, and induces the trans-differentiation of myoblasts into osteoblasts^10^. In addition, BMP-2 in solution plays a role in early adhesive events, including adhesion and migration through cytoskeletal reorganization^11^,^12^. Recently, several studies have demonstrated that BMP-2 strongly interacts with extra-cellular matrix (ECM) proteins^13^, especially fibronectin (FN) due to its highly promiscuous GF-binding site (the 12th to 14th type III repeats)^14^,^15^. Moreover, immobilized BMP-2 whether by physical adsorption (*i.e.* matrix-bound BMP-2^13^,^16^) or by covalent grafting^17^ was shown to regulate cell behavior quite distinctly from BMP-2 in solution. This effect is currently poorly known and is likely due to the close proximity and crosstalk of integrin-binding domains of FN and BMP-2^13^,^17^,^18^,^19^. It has indeed been shown that the secretion of FN by cells is necessary for BMP-2-mediated signaling^13^. A consequence of this association of BMP-2 with ECM proteins *in vivo* is that the spatially patterned presentation of BMPs by the ECM balances progenitor cell renewal and differentiation in the stem cell niche^20^,^21^,^22^,^23^ and guides numerous developmental and repair steps^24^,^25^,^26^.

Therefore, simple methods to recreate a niche microenvironment presenting specific mechanical properties, ECM proteins and locally immobilized GFs are important for elucidating fundamental features of developmental biology, as well as being a possible solution for guiding and enhancing tissue engineering approaches. Many ECM proteins have been patterned by microcontact printing on stiff substrates, aimed to study cell adhesion, migration, division and differentiation^27^,^28^,^29^,^30^. Wang *et al*. thus used FN micropatterns to demonstrate that the differentiation of human MSCs into osteoblasts in response to BMP-2 added in solution in the culture medium (*i.e.* soluble BMP-2) actually depends on cell shape and cytoskeletal tension^31^. In contrast, very few techniques exist for patterning GFs such as BMP-2 without chemically modifying the protein^32^. Recently, Cavalcanti-Adam *et al*. developed new approaches for immobilizing BMP-2, by using biotinylated BMP-2 or a heterobifunctional linker to covalently graft BMP-2 to a glass coverslip^33^,^34^. They demonstrated that immobilized BMP-2 triggered early differentiation signaling and increased migration of C2C12 myoblasts. However, chemical modifications and covalent immobilization of BMP-2 may hinder the biological activity of the BMP-2 or affect cell behavior^35^,^36^,^37^. Currently, the only existing methods for creating patterns of unmodified, matrix-bound BMP-2 on ECM coatings are based on inkjet printing. Campbell *et al*. thus used the natural affinity between BMP-2 and fibrin to generate 0.5–1 mm^2^ squares of immobilized BMP-2 on fibrin substrate^38^,^39^. They demonstrated the possibility to guide a stem cell population toward multiple fates on a single substrate and in a spatially defined manner. But the millimetric resolution of this approach limits its use to large, multi-cellular patterns, on which a part of the cell population responds to BMP-2 patterns differently than the rest of the population outside the patterns, independently of cell adhesion.

We previously reported that soft biopolymeric films composed of poly(L-lysine) (PLL) and hyaluronic acid (HA) can retain high and tunable quantities of BMP-2^40^. This interesting property was used to create large, centimeter-scale gradients of matrix-bound BMP-2 by microfluidics^41^. Moreover, the presentation of matrix-bound BMP-2 could rescue C2C12 myoblast adhesion on these otherwise non-adhesive soft biopolymeric films^16^ via a combined action between BMP receptors and integrin receptors^13^.

Here we built on these results to generate cellular- and sub-cellular-sized patterns of unmodified BMP-2 within a FN matrix on soft biopolymeric films. We demonstrate the ability to routinely produce arrays of FN-bound BMP-2 micropatterns with a micrometric resolution (∼3 μm). We examine the influence of the size of these BMP-2 micropatterns on cell spreading, cytoskeleton organization and early osteogenic trans-differentiation signaling with single-cell precision. This study demonstrates that the spatially patterned, FN-bound presentation of BMP-2 and the resulting cell spreading regulate the phosphorylation and translocation to the nucleus of SMAD1/5/8 in myoblasts, through the activation of LIM kinase (LIMK) via the Rho-associated kinase (ROCK) pathway. By associating microcontact printing and layer-by-layer (LbL) deposition, we were thus able for the first time to generate cellular- and subcellular-sized micropatterns of unmodified BMP-2 trapped in FN on soft biopolymeric films.

## Results

### Microcontact printing of BMP-2 and fibronectin on biopolymeric films

**Figure 1:**
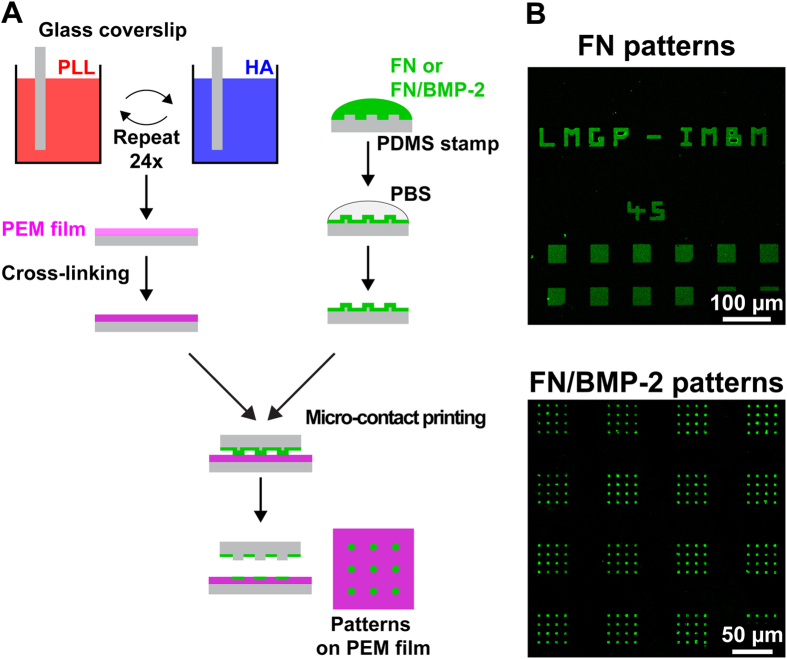
Microcontact printing of BMP-2 on soft biopolymeric films. (**A**) Schematic of the LbL buildup and crosslinking of (PLL/HA) films combined with microcontact printing (the ink containing either FN alone or BMP-2 within FN) for generating micropatterns of FN or FN/BMP-2 on soft (PLL/HA) films. (**B**) Representative images depicting the faithful reproduction of the photomask spatial features into FN (with 10% of fibrinogen^A488^) and FN/BMP-2^CF^ micropatterns.

We combined the techniques of LbL deposition and microcontact printing to create micropatterns of unmodified BMP-2 mixed with FN on soft biopolymeric films (Fig. 1). By alternatively dipping glass coverslips in PLL and HA solutions, we obtained (PLL/HA) multilayer films. (PLL/HA) films were then slightly cross-linked with a carbodiimide solution at 30 mg mL^−1^. To generate micropatterns of BMP-2, we first adsorbed a combination of BMP-2 and FN on a polydimethylsiloxane (PDMS) stamp before transferring this adsorbed layer of proteins onto a slightly cross-linked (PLL/HA) film. We thus obtained micropatterns of BMP-2-containing FN (hereafter called FN/BMP-2) on biopolymeric (PLL/HA) films (Fig. 1B), thanks to the very good affinity between BMP-2 and FN^13^,^14^,^15^. We also printed micropatterns of FN alone on (PLL/HA) films as negative (without soluble BMP-2) and positive (with soluble BMP-2, hereafter called sBMP-2) control conditions.

Of note, the use of a photoresist designed for high aspect ratios permitted a faithful reproduction of the photomask’s spatial features (Fig. 1B) and ensured a good, sub-cellular spatial resolution, as illustrated by the confinement of vinculin, a membrane-cytoskeletal protein involved in focal adhesions (FA), on micropatterns of ~3 μm diameter (Figure S1).

**Figure 2:**
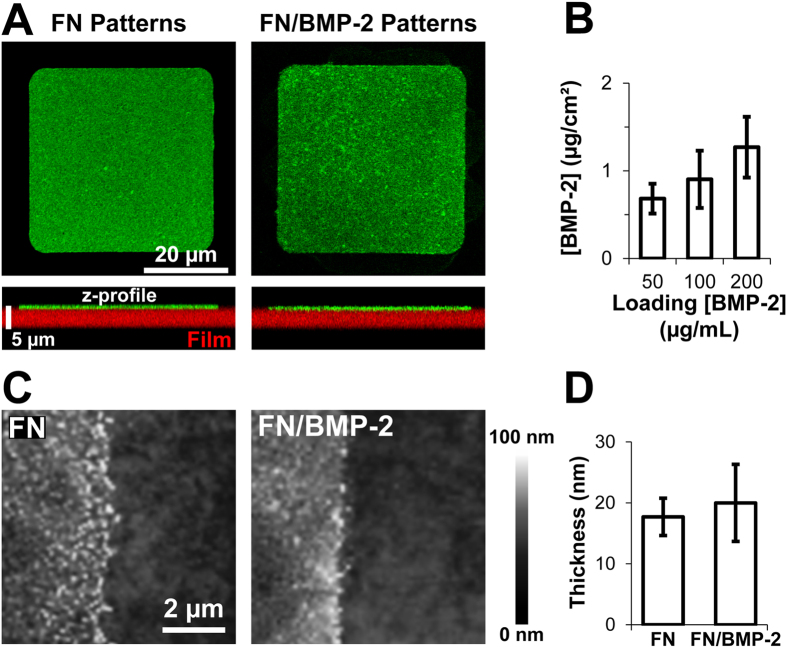
Characterization of the micropatterns. (**A**) Confocal images and cross-sections of micropatterns of FN (containing 10% of fibrinogen^A488^) and FN/BMP-2^CF^. The patterns are in green and the film in red (for the cross-section only). (**B**) Printed amounts of BMP-2 in micropatterns of FN/BMP-2 in function of the loading concentration of BMP-2 on the stamp. (**C**) High resolution AFM topography images and (**D**) thickness measurements of the protein layer on the border of micropatterns (located on the left of each image).

These slightly cross-linked (PLL/HA) films being soft, with a Young’s modulus ~200 kPa^42^,^43^, we first checked that they were not damaged during the microcontact printing (Fig. 2). A confocal view of micropatterned films cross-sections showed that the films were about 4 μm thick, with a very thin layer of proteins on top of it, and unmodified by the printing step (Fig. 2A). By combining fluorescence spectrometry and confocal imaging, we quantified the amount of patterned BMP-2 for different loading concentrations on the stamp (Fig. 2B). We measured a BMP-2 surface concentration from 0.7 ± 0.2 μg cm^−2^ to 1.3 ± 0.3 μg cm^−2^ when the loading BMP-2 concentration is increased from 50 to 200 μg mL^−1^ with a constant FN loading concentration of 50 μg mL^−1^. Note that all experiments were performed several hours after extensive rinsing of the patterned films so that only immobilized BMP-2 and FN remained. We verified that BMP-2 was not released from the micropatterns by quantifying the diffusion of printed BMP-2 labeled with 5(6)-carboxyfluorescein-N-hydroxysuccinimide ester (CF) by fluorescence recovery after photobleaching (FRAP) and its release in solution by fluorescence spectroscopy (Figure S2). No recovery of fluorescence was observed 4 h after photobleaching, indicating the absence of diffusion of BMP-2 when patterned within FN (Figure S2A). Similarly, we did not measure any significant fluorescence of BMP-2^CF^ in solution after 4 h at 37 °C, meaning that, if present, the BMP-2 concentration in solution was less than 8 ng mL^−1^ (Figure S2B), which is known to be too low to trigger osteogenic trans-differentiation^34^,^40^. As the z-resolution of laser scanning confocal microscopy is limited to ~500 nm, we also imaged micropatterns of FN/BMP-2 and FN by atomic force microscopy (AFM) (Fig. 2C). We thus confirmed that the films were not damaged during microcontact printing and we measured the thickness of the protein layers. FN/BMP-2 and FN patterns were both close to 20 nm thick, suggesting that BMP-2 dimers, with an estimated size of approximately 6.4 × 3.4 × 3.0 nm per dimer^34^,^44^, were buried within a mesh of FN strands, which were shown to be approximately 120 × 2 ×2 nm^45^.

By associating microcontact printing and LbL deposition, we were thus able for the first time to generate cellular- and subcellular-sized micropatterns of unmodified BMP-2 trapped in FN on soft biopolymeric films without damaging the films. Due to the affinity between BMP-2 and FN^13^,^14^,^15^, this presentation mode allows to potentiate the effect of BMP-2 and FN, in a manner close to *in vivo* conditions.

### C2C12 myoblasts adhere and respond specifically to FN-bound BMP-2 micropatterns

**Figure 3:**
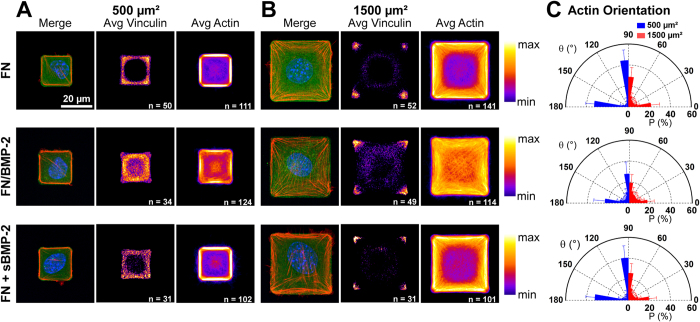
C2C12 myoblasts adhere and respond to micropattern geometry on soft biopolymeric films. Individual C2C12 myoblasts, average vinculin and average actin images over *n* cells on small 500 μm^2^ (**A**) and large 1500 μm^2^ (**B**) micropatterns of FN/BMP-2 and FN alone with and without soluble BMP-2 (sBMP-2) after 4 h of culture. Micropatterns are in green, actin in red and nuclei in blue. (**C**) Corresponding actin orientation.

We previously demonstrated that C2C12 myoblasts poorly adhere on slightly cross-linked (PLL/HA) films^13^,^16^. By microcontact printing patterns of FN/BMP-2 on these films, we thus generated cell-adhesive micropatterns on a non-cell-adhesive surface. Indeed, C2C12 myoblasts selectively adhered on both the FN/BMP-2 and the FN patterns, whereas almost no cells were observed outside of the patterns (Fig. 3 and Figure S3).

Previously, it had been shown that cell shape strongly regulates cell behaviors such as differentiation, mitosis or apoptosis^28^,^29^,^30^. In order to evaluate the impact of cell spreading and cytoskeletal tension on the signaling pathways induced by FN-bound BMP-2, we designed small, 500 μm^2^ micropatterns that roughly correspond to the size of attached but hardly spread C2C12 cells, and large, 1500 μm^2^ micropatterns matching the size of fully spread C2C12 cells^16^ (Fig. 3). We observed that C2C12 cells always spread over the whole FN/BMP-2 or FN pattern and match its shape. The ability to create single cell-size micropatterns thus allowed us to control cell spreading and subsequently cytoskeletal organization and tension. C2C12 myoblasts presented a very specific cytoskeletal organization on the micropatterns, with very thick actin fibers along the sides of square patterns on FN and FN/BMP-2 patterns. Moreover, we observed that in the case of patterns of FN-bound BMP-2, there was a significant recruitment of actin fibers around the nucleus when compared to FN patterns with or without sBMP-2 (Fig. 3 and Figure S4). 3D reconstruction of actin staining showed that C2C12 myoblasts on FN/BMP-2 patterns presented a specific and strong cytoskeletal continuity between peripheral stress fibers and the nucleus (Figure S5). We also performed immunofluorescent staining to observe the localization of vinculin. We noticed a good correlation between vinculin localization and actin stress fiber anchorage sites (Fig. 3A,B). Indeed, vinculin was more prominent on the outer edges of the FN patterns, in the presence or not of sBMP-2, whereas it was more homogeneously located over the entire area of the cell for patterns of FN/BMP-2, indicating a higher probability to generate adhesions sites over the entire cell surface in this specific condition. The quantification of actin orientation (Fig. 3C) revealed that about 75% of actin fibers were parallel to the square sides (*i.e.* with an angle <10° or >80°) on 500 μm^2^ FN squares, and 50% aligned along the sides of 1500 μm^2^ FN squares without sBMP-2. Actin organization was not affected by the presence of sBMP-2 and presented similar orientations, confirming the importance of BMP-2 presentation mode on cell behavior. For all conditions, the differences in relative actin orientation observed between the 500 and the 1500 μm^2^ squares are mainly due to the presence of radial actin stress fibers on the larger patterns, which reinforce the actin cytoskeleton to sustain the larger spreading of the cell^46^,^47^. We found less aligned actin fibers on FN/BMP-2 micropatterns, with percentages decreasing to 50% and 36% on small and large squares, respectively. This loss of actin alignment on BMP-2-containing patterns is mostly due to the emergence of numerous reinforcing fibers around the nucleus (Fig. 3 and Figures S4 and 5), confirming the vinculin staining.

Thanks to the contrast of cell-adhesive properties of the slightly cross-linked (PLL/HA) films and the FN, we could simultaneously control the spatial presentation of FN-bound BMP-2 and cell spreading, revealing the specific impact of this presentation mode of BMP-2 on the cytoskeletal continuity between peripheral stress fibers and the nucleus of C2C12 myoblasts.

### FN-bound BMP-2-induced SMAD signaling depends on cell spreading, through a LIM kinase dependent pathway

**Figure 4:**
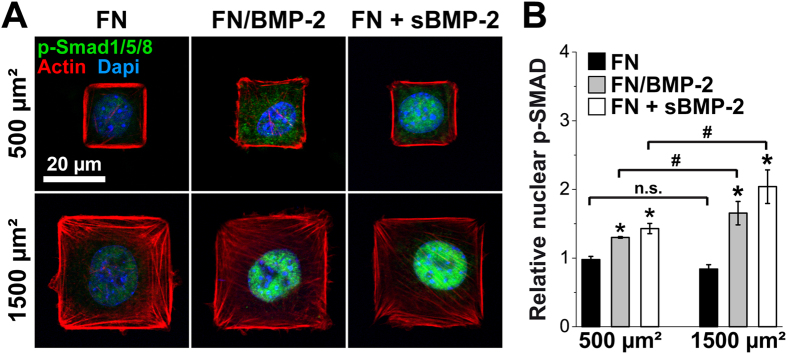
Phosphorylation and translocation of SMAD1/5/8 to the nucleus depends on cell spreading. (**A**) Immunofluorescence images of C2C12 myoblasts spread on small (500 μm^2^) and large (1500 μm^2^) micropatterns of FN/BMP-2 and FN without (negative control) or with (positive control) BMP-2 in solution after 4 h of culture. Actin is in red, nuclei in blue and p-SMAD1/5/8 in green. (**B**) Quantification of the relative nuclear p-SMAD1/5/8 (*n* > 100 cells) in function of the size and composition of the micropatterns. *p < 0.01 versus negative control (FN patterns without BMP-2 in solution); ^#^p < 0.01 between small and large micropatterns. n.s. stands for non-significant (i.e. p > 0.01).

As we observed a clear impact of FN-bound BMP-2 on cytoskeletal organization, especially around the nucleus, we next investigated whether this effect was correlated with BMP-2-dependent regulation of transcription factors (Fig. 4). The BMP-2 micropatterns are stable over time and are bioactive for at least 4 days, as proved by the expression of the osteogenic marker alkaline phosphatase (ALP)^10^ (Figure S6). Nevertheless, the present study aims at studying the short-term behavior of single cells confined by the micropatterns, whereas for the ALP study cells proliferated over 4 days of culture and we could only find very few micropatterns containing only a single cell. We thus focused on the phosphorylation and nuclear translocation of SMAD1/5/8 after 4 h of culture, which is a hallmark of the early signaling associated to C2C12 trans-differentiation^13^,^48^. Indeed, SMAD is a transcription factor known to play a key role in the transduction pathway from BMP-2 receptors to the nucleus^49^,^50^. We thus examined by immunofluorescence whether SMAD1/5/8 phosphorylation and translocation to the nucleus were impacted by FN-bound BMP-2 and cell spreading.

We first verified the specificity of the immunostaining of p-SMAD1/5/8 by examining its presence and location in C2C12 myoblasts spread on control FN patterns, without or with BMP-2 in solution (Fig. 4A). In the absence of BMP-2, we observed only a small amount of p-SMAD1/5/8 in the cytoplasm and nucleus of C2C12 myoblasts plated on both small and large FN micropatterns. In the presence of BMP-2 in solution, p-SMAD1/5/8 was highly enriched in the nuclei of myoblasts adhering on FN patterns. Furthermore, this amount of nuclear p-SMAD1/5/8 appeared to depend on cell area, increasing with the degree of cell spreading (Fig. 4B). The presentation of FN-bound BMP-2 also triggered the phosphorylation of SMAD1/5/8 and its translocation into the nucleus. Moreover, we observed similar effects of cell spreading on the phosphorylation of SMAD1/5/8 (Fig. 4A,B). The level of nuclear p-SMAD1/5/8 was significantly higher for myoblasts spread over large, 1500 μm^2^ patterns, compared to smaller, 500 μm^2^ squares.

We thus demonstrated that the presentation of BMP-2 by FN micropatterns was able to trigger the phosphorylation of SMAD1/5/8 and its translocation to the nucleus. For the first time, we demonstrated that BMP-2-induced osteogenic trans-differentiation signaling was also regulated by cell spreading when BMP-2 was bound to FN.

Of note, we observed a similar adaptation of the cell morphology to the pattern for two other cell types, namely mouse mesenchymal stem cells (D1 MSC) and human immortalized myoblasts (hMyoblasts) (Figure S7A). We measured an elevated amount of nuclear p-SMAD1/5/8 for both cell types, which was statistically significant for D1 MSC (Figure S7B). The impact of BMP-2 was lower in hMyoblasts, probably because even large 1500 μm^2^ micropatterns were not large enough to accommodate these cells.

**Figure 5:**
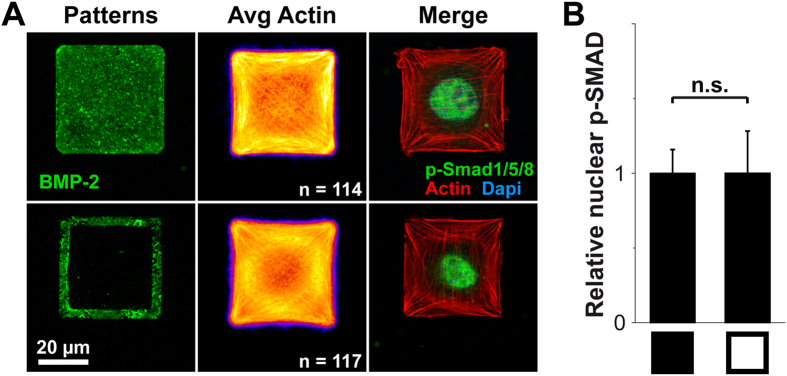
Spreading-dependent BMP-2-induced p-SMAD1/5/8 translocation is independent of the available amount of BMP-2. (**A**) Solid (top) and hollow (bottom) 1500 μm^2^ square micropatterns of FN/BMP-2 with BMP-2 in green, corresponding average actin images over *n* cells and immunofluorescence images of C2C12 myoblasts after 4 h of culture where actin is in red, nuclei in blue and p-SMAD1/5/8 in green. (**B**) Quantification of the relative nuclear p-SMAD1/5/8 for both conditions (*n* > 100 cells). n.s. stands for non-significant (i.e. p > 0.01).

On FN/BMP-2 micropatterns, the amount of BMP-2 presented to the cell is proportional to the pattern size. Thus, in order to confirm that the increase of the phosphorylation and translocation of SMAD1/5/8 when cells spread on large versus small BMP-2 patterns was indeed due to cell spreading, and not to the available amount of BMP-2, we compared the levels of p-SMAD1/5/8 in cells spread on solid versus hollow squares of FN/BMP-2 (Fig. 5). The available spreading area is the same for both conditions, even though the amount of available BMP-2 is lower on the hollow squares (about 1/3 of the BMP-2 amount of solid square patterns). Cells spread across the non-printed areas on hollow squares and presented similar spreading area and cytoskeleton organization than cells on solid patterns. The amount of nuclear p-SMAD1/5/8 was similar for both conditions (Fig. 5B). Thus, cell spreading appears to be determinant in regulating BMP-2-induced SMAD1/5/8 signaling.

**Figure 6:**
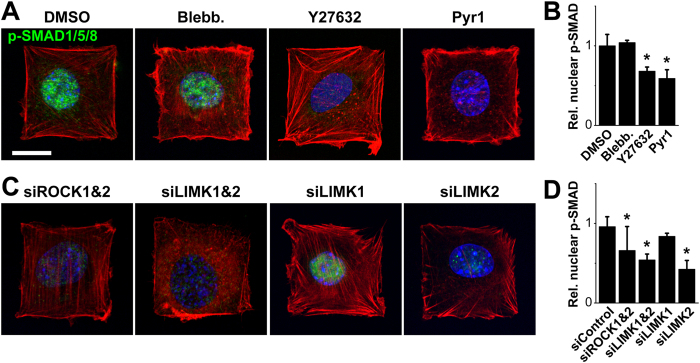
Early SMAD1/5/8 signaling induced by FN-bound BMP-2 depends on LIM kinase but not myosin II activity. (**A**) Immunofluorescence images and (**B**) quantification of the relative nuclear p-SMAD1/5/8 (*n* > 60 cells) of C2C12 myoblasts after 4 h of culture on 1500 μm^2^ square micropatterns of FN/BMP-2 in presence of DMSO, blebbistatin (Blebb), Y27632 or Pyr1. (**C**) Immunofluorescence images and (**D**) quantification of the relative nuclear p-SMAD1/5/8 (*n* > 60 cells) of C2C12 myoblasts depleted in ROCK1&2, LIMK1&2, LIMK1 or LIMK2 using a siRNA strategy. Actin is in red, nuclei in blue, and p-SMAD1/5/8 in green. Scale bar is 20 μm. *p < 0.01 versus control (i.e. DMSO or siControl).

To further our understanding of the potential role of the cytoskeleton in FN-bound BMP-2-induced SMAD1/5/8 signaling, we used a pharmacological and siRNA-mediated knockdown approach to interfere with cell tension and cytoskeleton dynamics (Fig. 6), and subsequently with BMP-2-mediated SMAD signaling^13^,^31^. Cell shape and spreading were unaffected by the inhibition of the motor protein myosin II by blebbistatin^51^, the inhibition of the Rho-associated kinase (ROCK) by Y27632^52^ or the inhibition of the ROCK effector LIM kinase (LIMK), which inhibits the actin-depolymerizing protein cofilin, by Pyr1^53^ (Fig. 6A). The amount of nuclear p-SMAD1/5/8 was unchanged by the inhibition of myosin II whereas the inhibition of ROCK and of LIMK induced a drastic and significant decrease of nuclear p-SMAD1/5/8 (Fig. 6B). The siRNA-mediated silencing of ROCK1&2 and LIMK1&2 confirmed the effects of the chemical inhibitors (Fig. 6C,D), both of them inducing a statistically significant diminution of nuclear p-Smad1/5/8 (Fig. 6D). As only LIMK2 is a downstream effector of ROCK^54^, we examined if each LIMK isoform could have different effects of SMAD. Specific siRNA knockdowns against LIMK1 and LIMK2 revealed that only LIMK2 silencing induced a strong decrease of the level of nuclear p-SMAD1/5/8, confirming the crucial role of the ROCK-LIMK2 pathway in the BMP-2-induced early SMAD signaling. The efficacy of the siRNA-mediated knock-down was confirmed by Western blots (Figure S8).

Of note, we obtained similar results with cells on FN patterns with sBMP2, with no effect of blebbistatin on the nuclear p-SMAD1/5/8 and a strong decrease with Y-27632, Pyr1, siROCK1&2 and siLIMK1&2 (Figure S9).

## Discussion

BMP-2 is a very potent morphogen playing an important role in morphogenesis and tissue homeostasis^6^,^7^, and appears to be a powerful inducer of osteogenic differentiation in MSCs^9^,^31^, osteoblasts^8^,^9^ and myoblasts^10^. Interestingly, although the mode of presentation of BMP-2 (*i.e.* in solution or matrix-bound) has been shown to distinctly regulate cellular responses^16^,^17^, the impact of a biomimetic, FN-bound presentation of BMP-2 on cell adhesion, cytoskeletal organization and BMP-2 signaling is still mostly unknown. We now show that the degree of cell spreading as well as the presentation of BMP-2 by an ECM protein can regulate actin organization and BMP-induced SMAD signaling, and in the case of FN does so through a LIM kinase activation. Given the key role of BMP-2 in developmental processes, the link between BMP-2 presentation mode, cytoskeletal organization and BMP-2 signaling may participate in specific osteogenic differentiation events during musculo-skeletal development. Moreover, understanding how matrix-bound BMP-2 regulates osteogenic differentiation is crucial for developing clinical therapies for critical bone defects^3^,^55^.

Taking advantage of the very good affinity between BMP-2 and FN^13^,^14^,^15^, we generated micropatterns of BMP-2 within FN on soft biopolymeric films. The potential of these LbL films loaded with BMP-2 for implant coatings was previously demonstrated^55^,^56^,^57^,^58^. (PLL/HA) films were chosen as the ability of cells to adhere and spread on these films strongly depends on the film’s cross-linking^43^,^59^ and/or the presence of matrix-bound BMP-2^13^,^16^. Slightly cross-linked (PLL/HA) films were thus previously shown to have lasting, stable non-cell-adhesive properties^43^,^59^, while cells adhere and rapidly spread on the same films containing matrix-bound BMP-2^13^,^16^. By microcontact printing patterns of FN/BMP-2 on these films, we thus generated cell-adhesive micropatterns on a non-cell-adhesive surface, with a sub-cellular spatial resolution, close to 3 μm (Figure S1), which is comparable to resolutions obtained for microcontact printing on stiff substrates^60^. Moreover, we avoided any protein modification which could affect the biological activity of the BMP-2^35^,^36^,^37^. The films were not damaged during the microcontact printing and BMP-2 was immobilized within a mesh of FN strands (Fig. 2). When we seeded C2C12 myoblasts on micropatterned films, we observed a very selective adhesion of the cells only on the micropatterns, with hardly any cell outside of the patterns (Fig. 3 and Figure S3).

Previously, it had been shown that cell shape strongly regulates cell behaviors such as differentiation, mitosis or apoptosis^28^,^29^,^30^. By designing small and large micropatterns that roughly correspond to the size of unspread C2C12 cells and fully spread C2C12 cells, respectively^16^, we could control and normalize cell spreading and, subsequently, cytoskeletal organization and tension (Fig. 3). C2C12 myoblasts presented a very specific cytoskeletal organization on the micropatterns, with very thick actin fibers along the sides of square patterns on FN and FN/BMP-2 patterns. The presence of FN-bound BMP-2 but not sBMP-2 induced a recruitment of actin fibers across the cell and around the nucleus, suggesting that the presence of BMP-2 within the FN, or the resulting proximity of BMP receptors and integrins^13^, regulates the cytoskeletal organization quite distinctly from free BMP-2 in solution. We demonstrated that BMP-2 mingled with FN was bioactive, as it triggered a significant translocation of p-SMAD1/5/8 into the nucleus (Fig. 4) and ALP activity (Figure S6). We showed that the amount of nuclear p-SMAD1/5/8 increased with the degree of cell spreading for both conditions of soluble and FN/bound BMP-2 (Fig. 4).

Our results are consistent with those obtained by Wang *et al*. for human MSCs cultured on stiff glass substrates in the presence of soluble BMP-2^31^. But contrary to this previous work, the available amount of BMP-2 in our study depends on the area of the micropatterns when BMP-2 is presented by the matrix. By comparing the levels of p-SMAD1/5/8 in cells spread on solid versus hollow squares of FN/BMP-2, we demonstrated that cell spreading was also determinant when BMP-2 was bound to the FN.

Recently, the presentation mode of BMP-2 was shown to influence cell behavior, as matrix-bound BMP-2 and covalently grafted BMP-2 were found to trigger stronger and more sustained effects than soluble BMP-2^13^,^16^,^17^. Wang *et al*. found that SMAD signaling, induced by soluble BMP-2 in human MSCs cultured on glass, was regulated by cytoskeletal tension via the ROCK pathway^31^. In contrast, Fourel *et al*. demonstrated using soft biopolymeric films and C2C12 cells that early SMAD signaling was dependent on the Cdc42/LIMK pathway and independent of ROCK^13^. Using micropatterns of FN-bound BMP-2 on a soft biopolymeric film, we showed that both ROCK and LIMK2 are involved in SMAD signaling, in a myosin II–independent manner (Fig. 6).

These slight discrepancies may be due to differences in experimental settings such as the cell type (starved human MSCs^31^ or non-starved C2C12 myoblasts^13^), the BMP-2 presentation mode (in solution^31^, adsorbed on LbL films^13^ or patterned with FN in the herein work), the underlying substrate (glass^31^ or soft biopolymeric film^13^) or the source of FN (provided by the cells^13^, microcontact printed alone^31^ or mingled with BMP-2 in the present study). As our study focused on early SMAD signaling at 4 h, we do not exclude the involvement of myosin II in FN-bound BMP-2-induced signaling at later stages of differentiation. Nevertheless, the role of LIMK in the phosphorylation and inactivation of cofilin supports the need for spatial and temporal control of actin organization to initiate or maintain the osteogenic commitment^13^,^61^. Since BMP-2 plays a ubiquitous role in regulating morphogenesis, this link between BMP-2 presentation mode, cellular mechanics and signal transduction may provide a broader model for better understanding the impact of the spatial presentation of growth factors by ECM proteins on tissue formation, organization and homeostasis.

In conclusion, we demonstrated the possibility for the easy and versatile generation of cellular- and sub-cellular-sized patterns of unmodified BMP-2 on soft biopolymeric films, combined to an ECM protein. We utilized these BMP-2 micropatterns to investigate the effect of FN-bound BMP-2 on myoblast cytoskeletal organization, and the subsequent effect of cell spreading on early SMAD 1/5/8 signaling. We observed that FN-bound BMP-2 triggered the phosphorylation of SMAD1/5/8 and its translocation to the nucleus. We showed that SMAD signaling induced by FN-bound BMP-2 was regulated by cell spreading and actin dynamics through a myosin II-independent, ROCK-LIMK2 pathway in myoblast cells. Altogether, our results open an exciting avenue for studying the mechanisms of integrin/BMP receptor crosstalk and mechanotransduction involved when BMP-2 is presented by the ECM. Thus, patterns of FN-bound BMP-2 on soft biopolymeric films could become a useful tool for studying the impact of matrix-bound BMP-2, from its interaction with the ECM to cell adhesion and differentiation, in reproducible and standardized conditions. Most importantly, our approach could be adapted to other combinations of ECM proteins and GFs, depending on their respective affinity^14^, providing valuable opportunities to recreate tissue-specific niche microenvironments *in vitro* and analyze stem cell renewal and differentiation in well-defined conditions.

## Methods

### Polyelectrolyte multilayer (PEM) film buildup

PEM deposition was performed using poly(L-lysine) hydrobromide (PLL, Sigma) at 0.5 mg mL^−1^, and hyaluronic acid (HA, Lifecore) at 1 mg mL^−1^ dissolved in a Hepes-NaCl buffer (0.15 M NaCl, 20 mM Hepes pH 7.4). The first bilayer of the film was deposited manually, and was comprised of a layer of polyethylenimine (PEI, Sigma) followed by a layer of HA. The (PLL/HA)_24_ film (*i.e.* film made of 24 (PLL/HA) bilayers) buildup was done with an automatic dipping machine (Dipping Robot DR3, Kierstein GmbH). The subsequent cross-linking was achieved as previously described^62^ by using 1-Ethyl-3-(3-Dimethylamino-propyl) carbodiimide (EDC, Sigma) at 30 mg mL^−1^ and N-hydrosulfosuccinimide (sulfo-NHS, Sigma) at 11 mg mL^−1^.

### Microcontact printing

Polydimethylsiloxane (PDMS) stamps were made by casting Sylgard 184 (Dow Corning) liquid prepolymer over a silicon master fabricated by deep UV photolithography of a positive resist designed for high aspect ratios (AZ TX 1311, Microchemicals). Upon curing, the elastomeric stamp was peeled off, washed with ethanol, and dried under nitrogen. Stamps were then coated with an ink containing either fibronectin alone (FN) or FN and BMP-2 (FN/BMP-2). FN ink was composed of 45 μg mL^−1^ of FN and 5 μg mL^−1^ of Alexa Fluor 488 labeled fibrinogen from human plasma (Fibrinogen^A488^, Life Technologies) for visualization purposes, diluted in deionized H2O at pH 5. FN/BMP-2 ink was composed of 50 μg mL^−1^ of FN from human plasma (Roche Diagnostics) and 50 μg mL^−1^ of recombinant human BMP-2 (Clinical Grade, Wyeth BioPharma), of which 36% were labeled with 5(6)-carboxyfluorescein-N-hydroxysuccinimide ester (CF; Boehringer, Mannheim)^40^, in deionized H2O at pH 5. After a thorough rinsing with phosphate buffered saline (PBS), the inked stamps were blown dry under nitrogen. Simultaneously, the cross-linked (PLL/HA) films were placed in deionized water for 30 min to remove any trace of salt. They were then gently blow-dried under nitrogen and placed in conformal contact with an inked PDMS stamp for 1 min. The film-coated coverslip was then thoroughly washed in PBS before cell deposition. For control experiments, soluble BMP-2 was used at 600 ng mL^−1^ in the culture medium.

### Characterization of the patterns

Confocal images and fluorescence recovery after photobleaching (FRAP) experiments were conducted with a Zeiss LSM 700 confocal laser scanning microscope (Zeiss) equipped with a 63x oil immersion objective. Confocal images of isolated patterns were used to quantify the surface concentration of transferred BMP-2^CF^ thanks to a calibration curve obtained by UV-visible spectrometry using a microplate reader (Infinite M1000, Tecan) on PEM films homogeneously loaded with BMP-2^40^. Fluorescence recovery after photobleaching (FRAP) experiments were conducted to evaluate the possible diffusion of BMP-2^CF^ within (PLL/HA) films. To this end, a 15 μm diameter circular region of interest (ROI) was bleached using the 488 nm laser diode and the recovery after photobleaching was followed over time. The fluorescence intensity of the ROI was normalized to that of a control region. The release of BMP-2 from the patterns was evaluated by measuring the amount of BMP-2^CF^ in the medium every 10 min over 4 h at 37 °C with a UV–visible spectrometer (Infinite M1000, Tecan).

AFM images were obtained in PBS in peak force tapping mode using an AFM BioCatalyst instrument (Bruker). Pyramidal silicon nitride cantilevers (MSNL probes, Bruker) with a spring constant of 0.07 N m^−1^ were used. The analysis of the topography of 5 patterns per condition was performed using Nanoscope analysis (Bruker).

### Cell culture and reagents

Murine C2C12 skeletal myoblasts (<15 passages, obtained from the American Type Culture Collection, ATCC) were cultured in a 1:1 Dulbecco’s Modified Eagle Medium (DMEM):Ham’s F12 medium (Gibco, Invitrogen) supplemented with 10% FBS.

Murine D1 MSCs (<15 passages, obtained from the American Type Culture Collection, ATCC) were cultured in Minimum Essential Medium Eagle Alpha Modification medium (αMEM, Sigma Aldrich) supplemented with 10% fetal bovine serum FBS.

Human myoblasts were immortalized from a clone of primary human muscle stem cells isolated from a 53-year-old male individual, provided by the Institute of Myology (Paris) and cultured in a growth medium containing DMEM:Medium 199 (4:1) supplemented by 25 μg/ml fetuin, 5 μg/ml insulin, 0.2 μg/ml dexamethasone, 0.5 ng/ml basic FGF, 5 ng/ml human EGF, 20% FBS. All culture media were supplemented with 100 U mL-1 penicillin G and 100 μg mL-1 streptomycin (Gibco, Invitrogen).

Cells were deposited on the printed PEM film at a density of 10^4^ cells per cm^2^. Myosin II inhibitor blebbistatin (Calbiochem), LIM kinase inhibitor Pyr1 (kindly provided by Laurence Lafanechère, IAB, Grenoble) and ROCK inhibitor Y-27632 (Calbiochem) were introduced in the growth medium at a 20 μM^51^, 25 μM^53^ and 10 μM^31^ concentration, respectively.

### Cell staining

After 4 h of culture on the micropatterns, cells were fixed with 3.7% formaldehyde in PBS, permeabilized with 0.2% Triton X-100 in TBS (50 mM Tris-HCl, 0.15 M NaCl, pH 7.4) and blocked with 2% BSA (Aurion) in TBS. The samples were then incubated with primary antibodies against p-SMAD1/5/8 (Cell Signaling) or vinculin (Sigma Aldrich) and detected with Alexa 647- or Alexa 488-conjugated, isotype-specific, anti-IgG antibodies (Invitrogen). Actin was labeled with phalloidin-TRITC (Sigma) and nuclei were stained with DAPI (Life Technologies).

ALP was stained with fast blue RR salt in a 0.01% (w/v) naphthol AS-MX solution (Sigma Aldrich) according to the manufacturer’s instructions.

Image averaging and p-SMAD1/5/8 quantification were performed using home-made Image J (National Institutes of Health) routines. Briefly, nuclear and cytoplasmic p-SMAD1/5/8 fluorescence intensities were measured over nucleus and cytoplasm areas, respectively, obtained from binarized nucleus and actin images. The relative amount of nuclear p-SMAD1/5/8 was obtained by subtracting the cytoplasmic intensity from the nuclear intensity.

Actin orientation was evaluated with the Directionality plug-in (http://fiji.sc/wiki/index.php/Directionality) in Image J.

### siRNA interference

Cells were transfected with siRNA against LIMK1&2 or ROCK1&2 (ON-TAR GET plus SMARTpool) as previously described^13^. Briefly, cells were incubated with a transfection mix of Lipofectamine RNAiMAX Reagent (Invitrogen) and siRNA in Opti-MEM medium (Gibco) for two consecutive 24 h periods before seeding on patterned PEM films.

### Immunoblotting

Cells were lysed in Laemmli buffer. Detection of proteins by Western blotting was done according to standard protocols. After electrotransfer and blocking (10 mM Tris, pH 7.9, 150 mM NaCl, 0.5% Tween 20, and 3% dry milk at RT for 1 h), the PVDF membrane was incubated with antibodies overnight at 4 °C. Immunological detection was achieved with HRP-conjugated secondary antibody. Peroxidase activity was visualized by ECL (West pico signal; Thermo Fisher Scientific) using a ChemiDoc MP imaging system (Bio-Rad Laboratories). As control, detection of actin or GADPH was also performed.

### Statistical analysis

For each histogram, the mean represents the average of three to five independent experiments ± standard deviation. Significances were assessed by one-way analysis of variance, using Tukey’s honestly significant difference test to compare each pair of data.

## Additional Information

**How to cite this article**: Fitzpatrick, V. *et al*. Signal mingle: Micropatterns of BMP-2 and fibronectin on soft biopolymeric films regulate myoblast shape and SMAD signaling. *Sci. Rep.* **7**, 41479; doi: 10.1038/srep41479 (2017).

**Publisher's note:** Springer Nature remains neutral with regard to jurisdictional claims in published maps and institutional affiliations.

## Supplementary Material

Supplementary Information
