# Supplementary material for: Signal mingle: Micropatterns of BMP-2 and fibronectin on soft biopolymeric films regulate myoblast shape and SMAD signaling
Source: Sci Rep. 2017 Jan 30;7:41479. doi: 10.1038/srep41479 (PMC5278375; doi:10.1038/srep41479)
Supplement: Supplementary Information [file srep41479-s1.pdf]

Supporting Information

Signal mingle: Micropatterns of BMP-2 and fibronectin on soft biopolymeric films regulate myoblast shape and SMAD signaling.

*Vincent Fitzpatrick, Laure Fourel, Olivier Destaing, Flora Gilde, Corinne Albigès-Rizo, Catherine Picart and Thomas Boudou**

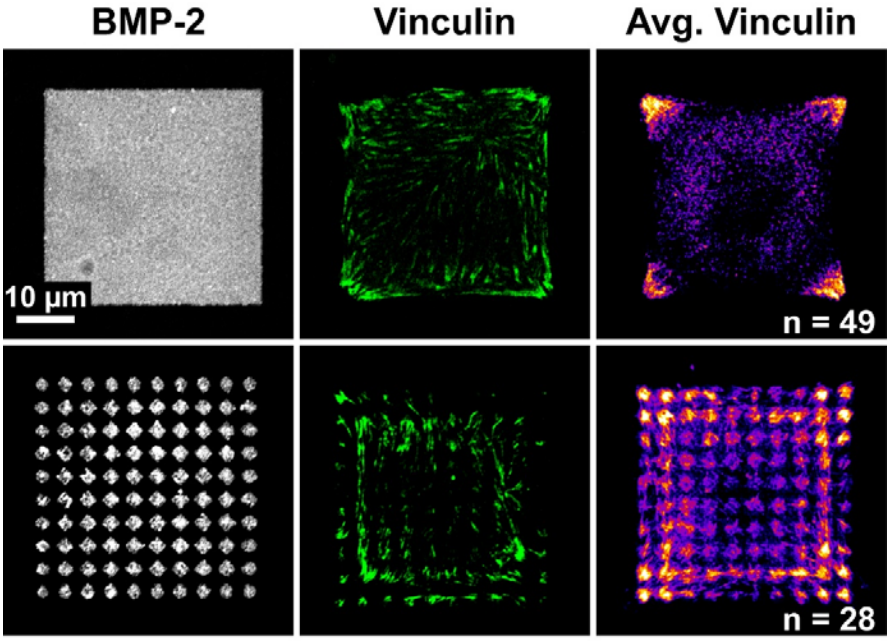

Figure S1. Sub-cellular micropatterns. Representative images of cellular (top row) and sub-cellular (bottom row, dot average diameter of 2.9 ± 0.3 μm.) micropatterns of FN/BMP-2 (in gray), with corresponding representative immunostainings of vinculin and average vinculin images over *n* cells.

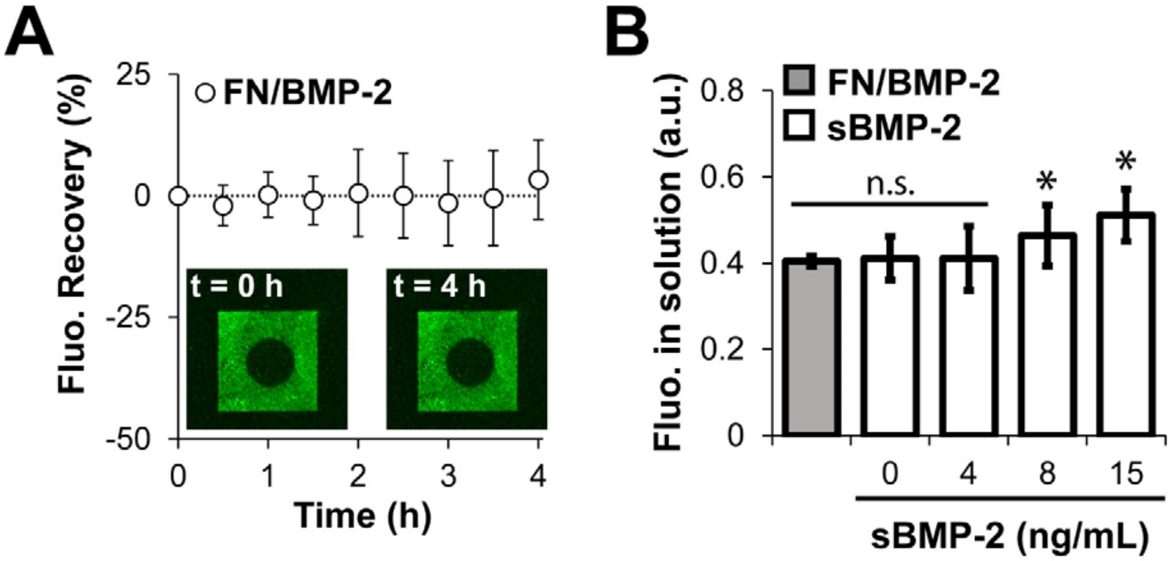

Figure S2. BMP-2 is immobilized when microprinted within FN. (A) Fluorescence recovery after photobleaching of BMP-2^{CF} in a FN/BMP-2 micropatterns over 4h. (B) Fluorescence of BMP-2^{CF} released in solution from FN/BMP-2 (dark gray) micropatterns after 4h at 37°C, compared with sBMP-2^{CF} at concentrations ranging from 0 to 15 ng mL^{-1} (light gray). * $p < 0.05$ versus negative control (no sBMP-2).

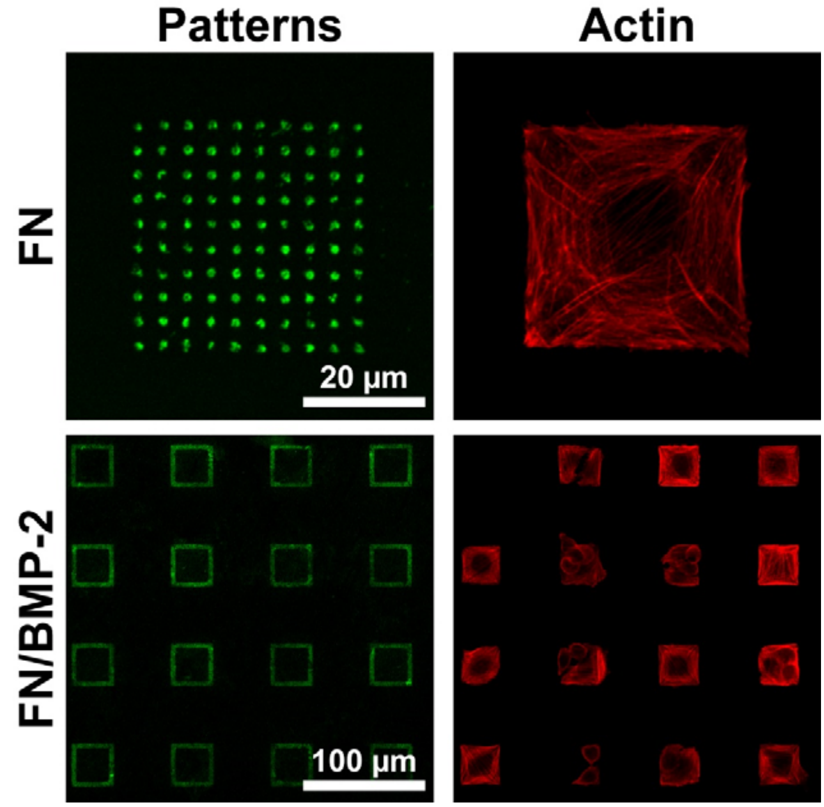

Figure S3. Selective adhesion of C2C12 myoblasts on the patterns. Representative images of C2C12 myoblasts (actin staining in red) on FN and FN/BMP-2 patterns (in green), highlighting the very selective adhesion of the cells on the patterns, whereas no cells adhere outside of the patterns.

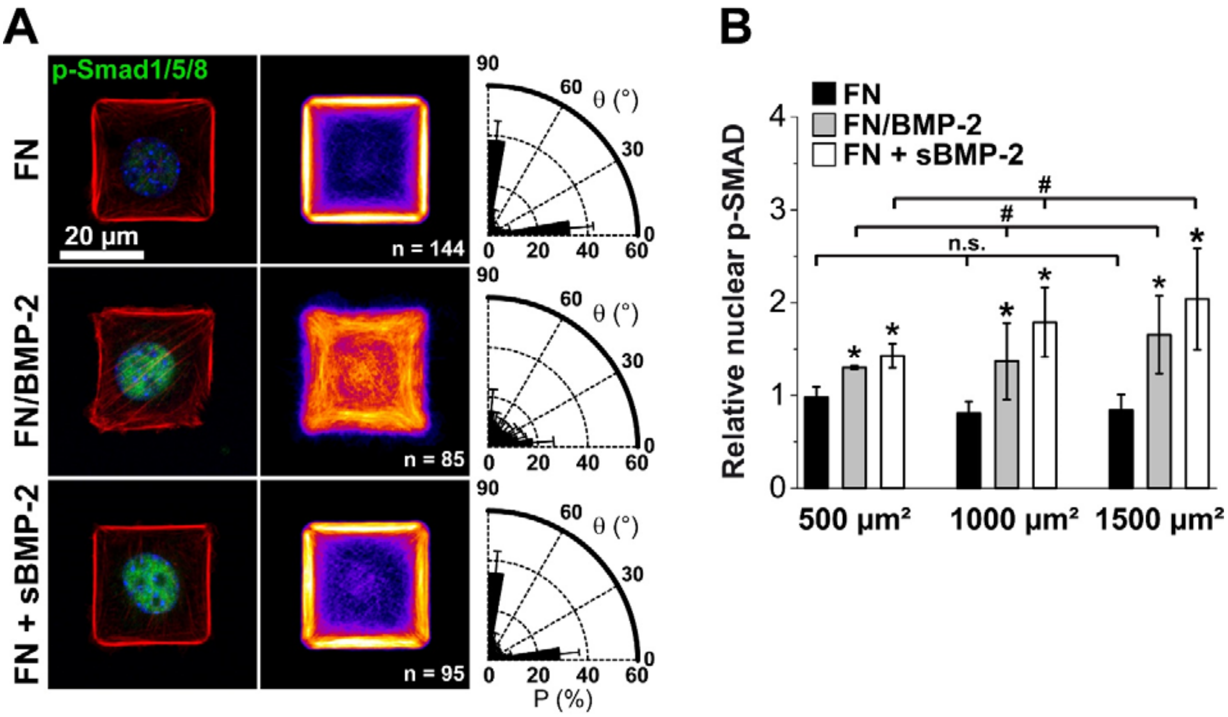

Figure S4. Response of C2C12 myoblasts on intermediate 1000µm² micropatterns (A) Individual C2C12 myoblasts, average actin images over *n* cells and corresponding actin orientation on intermediate 1000 µm² micropatterns of FN/BMP-2 and FN alone with and without sBMP-2 after 4 h of culture. Actin is in red, nuclei in blue and p-SMAD1/5/8 in green. (B) Quantification of the relative nuclear p-SMAD1/5/8 (n > 80 cells) in function of the size and composition of the micropatterns. * $p < 0.01$ versus negative control (FN patterns without BMP-2 in solution); # $p < 0.01$ between small, intermediate and large micropatterns. n.s. stands for non-significant (i.e. $p > 0.01$).

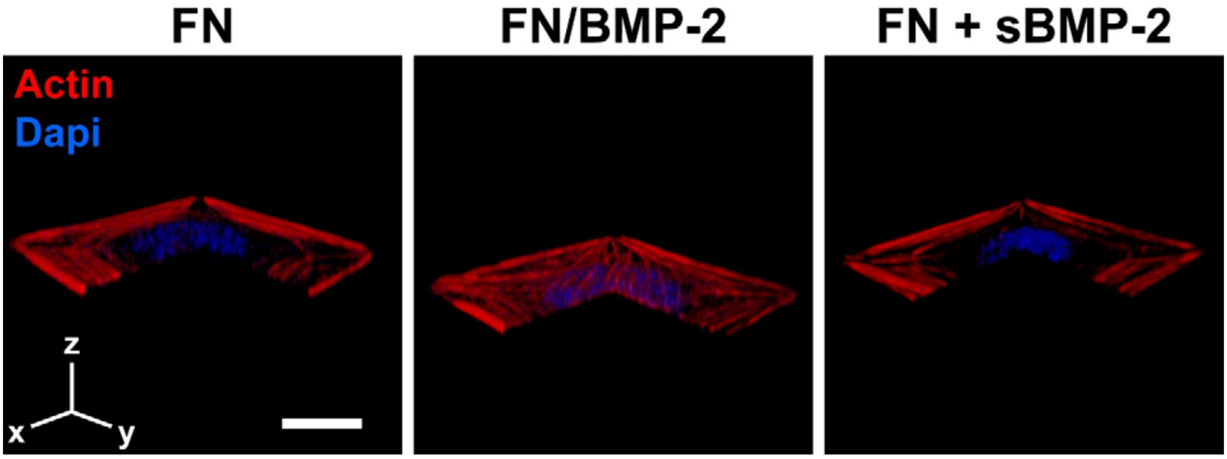

Figure S5. 3D actin organization. Representative 3D reconstructions of C2C12 myoblasts (actin in red, nucleus in blue) on 1500 µm² square micropatterns of FN/BMP-2 and FN without or with sBMP-2, highlighting the specific actin organization around the nucleus only for cells on micropatterns containing BMP-2. Scale bar is 10µm.

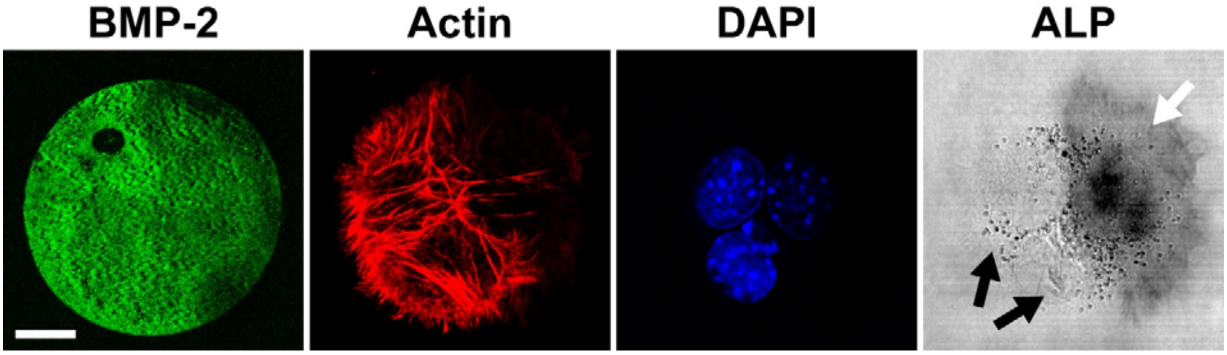

Figure S6. Long term culture and trans-differentiation of C2C12 myoblasts on FN/BMP-2 micropatterns. Representative images of C2C12 myoblasts cultured on FN/BMP-2 (in green) micropatterns for 4 days. Actin is in red, nuclei in blue and the histochemical staining of ALP is dark in the positive cell (white arrow) and non-apparent in the two negative cells (black arrows). Note that the fluorescence of the positive cell is absorbed by the ALP staining. Scale bar is 10 µm.

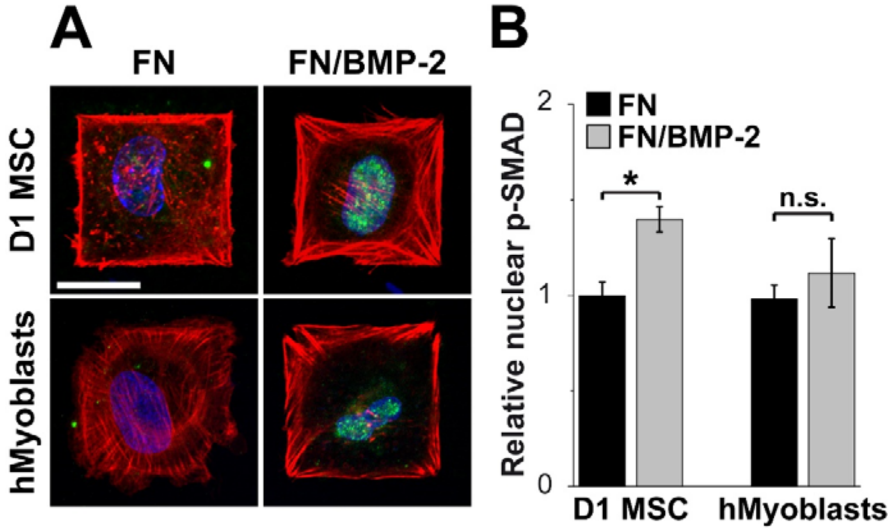

Figure S7. Phosphorylation and translocation of SMAD1/5/8 to the nucleus of D1 mesenchymal stem cells and human myoblasts. (A) Immunofluorescence images of D1 mesenchymal stem cells (D1 MSC) and human myoblasts (hMyoblasts) spread on large (1500 µm²) micropatterns of FN alone (negative control) and FN/BMP-2 after 4h of culture. Actin is in red, nuclei in blue and p-SMAD1/5/8 in green. (B) Quantification of the relative nuclear p-SMAD1/5/8 in function of the composition of the micropatterns for S1 MSC (*n* > 60 cells) and hMyoblasts (*n* > 20 cells). * $p < 0.01$ versus negative control (FN patterns without BMP-2 in solution); n.s. stands for non-significant (i.e. $p > 0.01$).

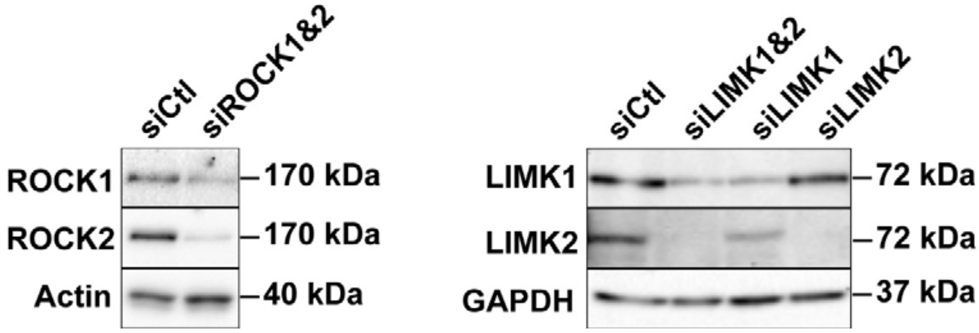

Figure S8. Efficiency of siRNA-mediated knock-down of ROCK1&2 and LIMK1&2. Western blot analysis confirms the efficiency of the siRNA against ROCK1&2, LIMK1&2, LIMK1 and LIM2.

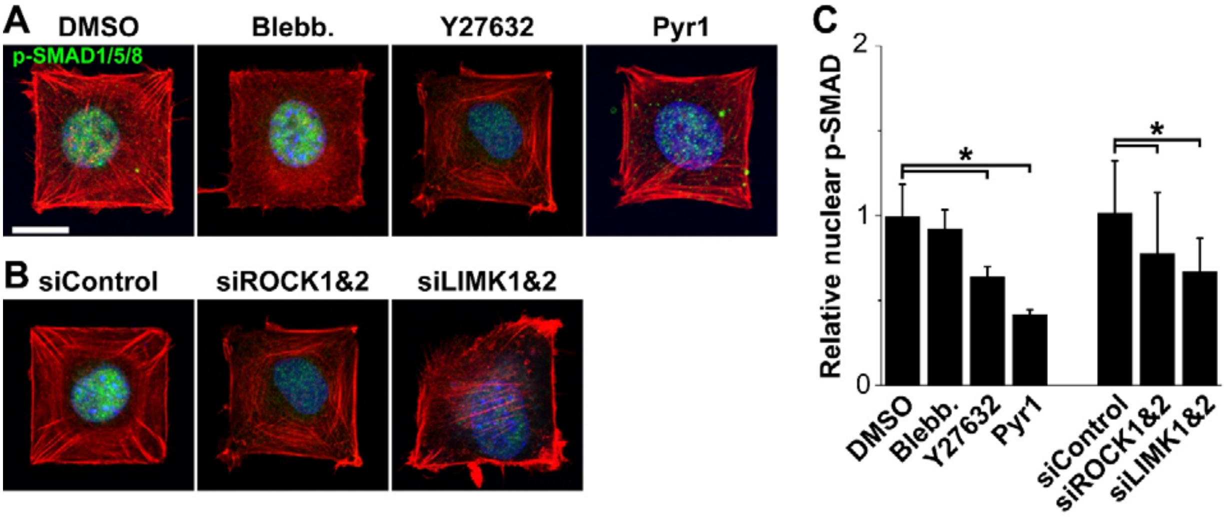

Figure S9. Early SMAD1/5/8 signaling induced by soluble BMP-2 depends on LIM kinase but not on myosin II. (A) Immunofluorescence images of C2C12 myoblasts after 4 h of culture on 1500 μm² square micropatterns of FN with sBMP-2 in presence of DMSO (control), blebbistatin (Blebb), Y27632 or Pyr1. Actin is in red, nuclei in blue, and p-SMAD1/5/8 in green. (B) Immunofluorescence images of C2C12 myoblasts after siRNA-mediated knockdown in ROCK1&2 or LIMK1&2 using siRNA strategy. (C) Quantification of the relative nuclear p-SMAD1/5/8 ($n > 60$ cells). Scale bar is 20μm. * $p < 0.01$ versus control (i.e. DMSO or siControl).
